# The Influence of Nonlinear Pedagogy Physical Education Intervention on Cognitive Abilities in Primary School Children: A Preliminary Study

**DOI:** 10.3390/brainsci15121283

**Published:** 2025-11-28

**Authors:** Elisa Pugliese, Pasqualina Forte, Carmela Matrisciano, Fabio Carlevaro, Cristiana D’Anna, Daniele Magistro

**Affiliations:** 1 Center for Neuroscience, University of Camerino, 62032 Camerino, Italy; elisa.pugliese@unicam.it (E.P.); pasqualina.forte@unicam.it (P.F.); carmela.matrisciano@univr.it (C.M.); cristiana.danna@unipegaso.it (C.D.); 2 Department of Education and Sport Sciences, Pegaso University, 80143 Naples, Italy; 3 Department of Neuroscience, Biomedicine and Movement, University of Verona, 37129 Verona, Italy; 4 Asti Higher Studies University Pole, Uni-Astiss, 14100 Asti, Italy; fabio.carlevaro@unito.it; 5 School of Health Science, Faculty of Environmental & Life Sciences, University of Southampton, Southampton SO17 1BJ, UK

**Keywords:** attention, developmental age, ecological-dynamic approach, physical activity, school-based intervention, speed processing

## Abstract

Background/Objectives: The study aims to experiment with a teaching methodology based on the application of some principles of Nonlinear Pedagogy (NLP) in order to understand its effectiveness not only on motor development but also on attention and processing speed. Methods: A between-subjects quasi-experimental design involved 165 children (mean age = 7.21 ± 0.93 years), assigned to an experimental (*n* = 98; 45% Male and 55% Female) and control group (*n* = 67; 42% Male and 58% Female) over 16 weeks (32 sessions). The experimental group followed Physical Education (PE) lessons grounded on NLP principles, while control group followed traditional PE lessons. Divided attention and visual processing speed were assessed using the Witty SEM test with 2 difficulty levels, and the motor skills were assessed through Test of Gross Motor Development-3 and used as covariate. Results: Significant interactions emerged for Divided Attention (*p* = 0.014, d = 58 for level 1; *p* = 0.014, d = 42 for level 2). The visual processing speed also showed significant interaction (*p* < 0.001, d = 0.88 for level 1; *p* < 0.001, d = 1.11 for level 2). Conclusions: Findings from this preliminary study indicate a significant relationship between NLP-based teaching and improvements in attention and visual processing speed. The NLP intervention group outperformed the control group in both domains, supporting the effectiveness of this pedagogical approach within primary school PE settings. These promising results encourage further investigation with larger samples and over longer intervention periods.

## 1. Introduction

During Childhood, development is characterised by profound biological, psychosocial, and emotional changes that support the emergence of higher cognitive functions. In this dynamic period, psychomotor development plays a foundational role through active interaction with the environment, children acquire and refine motor skills, which in turn stimulate neurocognitive processes [1,2,3,4,5]. 

Cognitive development refers to the evolving capacity to acquire, organize, and interpret information supporting abstract reasoning, decision-making, and environmental understanding [6,7]. Central to this evolution are executive functions, namely response inhibition, working memory, and cognitive flexibility, which underpin attentional control, academic performance, social interaction, and self-regulation [2,8,9,10]. The scientific literature has progressively examined the link between motor activity and cognitive development in childhood, with a particular focus on how structured and varied physical activity can enhance executive functioning, attention, and memory [11,12,13]. Importantly, interventions designed to involve children in stimulus-rich and variable movement contexts have shown potential in promising such cognitive outcomes [14,15,16]. These activities highlight the value of integrating movement and cognition in primary school context.

A particular theoretical framework to promote holistic child development in physical education is Nonlinear Pedagogy (NLP). In contrast to traditional approaches, which typically are founded on repetition and prescriptive instruction, NLP develops from the ecological dynamics approach and emphasises exploratory learning, adaptability to variable constraints, and learner autonomy. Within this framework, learning is considered as a process of self-organization, in which movement patterns emerge through the interaction of three aspects: individual, task, and environmental [17,18,19]. Recent school-based research reports positive outcomes associated with NLP-based interventions. For example, a three-month NLP programme implemented with fourth-grade students resulted in greater improvements in motor and cognitive creativity compared to traditional instruction, as measured by fluency, originality, flexibility, and problem-solving that involved constraint manipulation and variability [20]. Similarly, NaeimAbadi & Bagheri [21] demonstrated that NLP applied to obstacle race activities with female students enhanced physical literacy, motor competence, and functional variability, highlighting the adaptability and customisation offered by this approach. A meta-analysis comparing linear and nonlinear pedagogies in primary school physical education further highlighted the validity of NLP in promoting motor competence [22]. These results are strongly associated with the fundamental principles of NLP, such as the coupling perception-action, the manipulation of constraints and the functional variability to encourage exploration and adaptation. NLP allows children to actively construct solutions, rather than passively receiving instructions, promoting a learning environment rich in variability, challenges and autonomy [18,23,24]. Significantly, this pedagogical model aligns closely with the development of executive functions, including response inhibition, working memory, and cognitive flexibility. Because NLP requires children to continuously make decisions, adjust their behaviours, and react to dynamic and unpredictable task conditions, it naturally integrates motor, cognitive, and emotional requests. This integration supports the development of higher-order cognitive processes and contributes to enhanced self-regulation, attention, and problem-solving skills, key components of academic and social success during the early school years [25,26]. 

Emerging literature highlights the interdependence between cognitive and motor development, showing that both follow parallel trajectories during childhood and benefit from enriched learning environments [27,28]. Indeed, atypical or delayed development in one domain is often mirrored by deficits in the other [29]. As such, understanding the factors that support the concurrent growth of cognitive and motor capacities is of critical interest, particularly during early childhood when neural plasticity is high [30]. This neurocognitive enhancement is particularly evident during childhood, a period characterised by the strengthening and reinforcement of neural connections in response to task demands and environmental challenges. The specific characteristics of the activity are critical determinants in enhancing the cognitive benefits derived from physical exercise. According to Diamond [31], physical tasks that are cognitively demanding, emotionally engaging, and socially interactive are most effective. These characteristics align with the principles of Nonlinear Pedagogy, which emphasises variability, problem-solving, and adaptability in movement experiences. In such environments, children are required to make continuous decisions, regulate their emotions, and coordinate responses based on the changing demands of tasks, all of which actively engage executive processes [32,33]. Furthermore, the inclusion of variability in practice, such as modifying rules, task constraints, or environmental features, can foster the development of more advanced motor strategies and flexible cognitive control.

In the school context, the ecological-dynamic approach has emerged as an effective theoretical framework for promoting the development of cognitive functions through movement, and although experimental applications of Nonlinear pedagogy remain more frequently explored in the sports domain, they are increasingly being considered within educational practice as well. This is due to its emphasis on the dynamic interaction between the individual, the environment, and the task [34,35,36,37]. Based on the integration of perceptual ecology [38] and dynamic systems theory [39], the ecological-dynamic model provides a solid foundation for designing physical education (PE) activities that promote adaptability, motor problem solving, and cognitive flexibility, key components of meaningful and lasting learning during childhood [23,40]. In this perspective, motor behaviour is conceived as the emerging result of a complex, self-organised system that is continuously shaped by the interaction of various constraints. These constraints derive from individual characteristics (e.g., physical, cognitive, emotional), specific task requirements and characteristics of the surrounding environment [41]. Consequently, learning is not linear or prescriptive, but rather adaptive and contextual, built upon active engagement and discovery.

NLP represents a practical extension of the ecological-dynamic approach, offering a methodological framework that makes these theoretical principles operational in educational contexts. NLP promotes learning through exploration, adaptation, and autonomy, through the manipulation of constraints, the design of representative tasks, and the use of affordances, i.e., the opportunities for action perceived in the environment in relation to the student’s abilities [24]. From this perspective, learning is a nonlinear process shaped by the variability of movement and the coupling between perception and action. Game-based learning, as a preferred pedagogical strategy, improves motivation, emotional engagement and cognitive activation, thus promoting holistic development. Within this model, the teacher guides students through the adjustment of constraints to stimulate adaptive motor responses in meaningful and developmentally appropriate contexts [42,43,44]. The primary objective of this study is to evaluate the effectiveness of a school-based intervention delivered during physical education lessons, designed according to the principles of Nonlinear Pedagogy and the ecological-dynamic approach. The intervention seeks to promote stimulating, exploratory, and play-centred motor experiences, aimed at enhancing cognitive development in school-age children. Specifically, the study aims to determine whether such an approach can lead to measurable improvements in executive functions, with particular attention to attentional processes and processing speed, defined here as the brain’s capacity to quickly analyse stimuli, generate effective responses, and retain relevant information. By embedding cognitive challenges within physically and socially engaging contexts, the intervention aspires to support both motor and cognitive growth, contributing to a more integrated educational experience.

## 2. Materials and Methods

A between subject quasi-experimental design was adopted for this study; 165 first-year primary school children from a primary school were recruited. To be included, children had to attend physical education classes at school twice a week (for a total of two hours per week), as verified by school records. Eight first-grade classes were randomly allocated to the experimental or control group using a simple randomisation procedure (Microsoft Excel random number generator). Because the allocation occurred at the class level within a single school and teachers necessarily knew which activities they delivered, allocation concealment and blinding were not feasible in this educational setting. Participants were assigned to either an experimental group, which took part in the intervention, or a control group, which continued with standard physical education activities. The effectiveness of the intervention was assessed using a pre-test (T1) and a post-test (T2), administered before and after a 16-week intervention period. The intervention was delivered during regular school hours within the physical education curriculum and was grounded in the core principles of NLP. Each session was carefully structured to include the acquisition or consolidation of fundamental motor skills through activities that emphasised the integration of information and action, the manipulation of constraints and functional variability. These elements were purposefully designed to foster cognitive engagement, stimulate attentional control, enhance processing brain speed and promote the development of decision-making skills. Activities were delivered through play-based and exploratory formats, in accordance with the NLP approach, encouraging participants to engage in problem-solving and adapt to varied task demands. The manipulation of task, environmental, and individual constraints was employed to elicit functional movement solutions within dynamic and game-like contexts, aiming to enhance both motor competence and executive functioning through embodied learning experiences [24,42,45].

### 2.1. Participants

**Figure 1:**
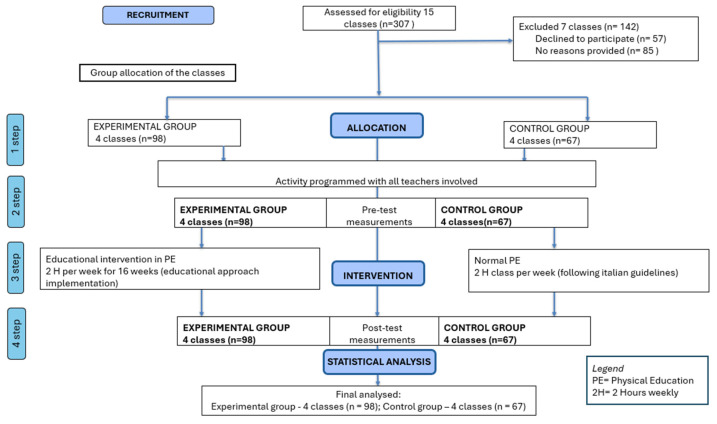
Participant Flow.

Eight first-grade classes from a primary school in southern Italy participated in the study, yielding a total sample of 165 children (M = 7.21, SD = 0.93). Participants were allocated into a control group (CG; *n* = 67) and an experimental group (EG; *n* = 98) based on class membership, following a convenience sampling approach aligned with school organisational constraints. Only the experimental group received the intervention. Figure 1 represent a flow diagram of participant, recruitment, allocation, intervention and analysis following CONSORT recommendations [46]. 

Based on the final sample (experimental = 98; control = 67) and an ANCOVA framework with baseline as a covariate (α = 0.05, power = 0.80), the unadjusted minimum detectable effect size (MDES) was *d* ≈ 0.43. Assuming a pre–post correlation of *r* = 0.60, the adjusted MDES was *d* ≈ 0.35–0.36. Accounting for potential class-level clustering (design effect = 1 + [*m* − 1] × ICC, with ICC = 0.02–0.05 and average class size *m* = 15–20), the cluster-adjusted MDES ranged from *d* ≈ 0.40 to 0.50.

The intervention was delivered by a graduate in Sports Science under the supervision of the classroom teacher, who was also responsible for delivering physical education in accordance with the Italian national curriculum. This ensured that both the pedagogical and curricular aspects of the intervention aligned with the National Curriculum Guidelines [47], which emphasise the development of body awareness, motor and postural control, and the ability to adapt to the spatial and temporal dynamics of the surrounding environment.

According to the Specific Learning Objectives for the End of Primary School Cycle (Grade 3) outlined in the National Guidelines, the motor dimension focuses on acquiring and developing fundamental motor skills, performing basic tactics and abilities applicable in games, and progressively enhancing body expression and communication. The cognitive dimension emphasises memorising and understanding rules and tactics, fostering a positive relationship with the learning environment, and promoting knowledge of healthy lifestyle principles.

While the EG participated in the Nonlinear Pedagogy-based programme, the CG followed the standard physical education curriculum, as specified by the Italian Ministry of Education [47,48], without any methodological modifications. In the CG, a traditional, prescriptive, and linear teaching approach was adopted, with motor skill acquisition based on repetitive, part-practice drills rather than integrated or global activities. The CG sessions were also delivered by a graduate in Sports Science under the supervision of the classroom teacher.

### 2.2. Intervention

The intervention was carried out during regular physical education lessons and followed a structured methodology grounded in the principles of NLP. It spanned a period of 16 weeks, with two sessions per week, amounting to a total of 32 sessions. Each session lasted approximately 50 min and followed a consistent triadic structure, comprising an initial phase (activation/warm-up), a central phase (cognitive motor), and a final phase (cool-down/reflection).

Fidelity of implementation was systematically monitored throughout the 16-week program using teacher logs, attendance records, and deviation reports. A total of 100% of the planned 32 sessions) were delivered as scheduled. The mean participant attendance rate across the intervention period was 95.4% (22.9 out of 24 sessions on average per child). Fidelity was supervised by trained teachers, external observers every two weeks, and the research team, who maintained detailed records and verified adherence using a structured checklist based on the nonlinear pedagogical principles (task variability, manipulation of constraints, and exploration time).

Throughout the 16-week intervention, all sessions were supervised by trained physical education teachers and at least one member of the research team. A standardised incident log was used to record any potential adverse events (e.g., injuries, discomfort, or excessive fatigue) occurring during or immediately after the sessions. Teachers were instructed to report all incidents to the research team, who reviewed reports weekly. No serious or minor adverse events were reported during the study period.

The activities were delivered in dynamic, variable contexts, designed to stimulate motor learning, cognitive engagement, and social interaction. The overarching aim of the intervention was to foster integrated development across the physical, cognitive, emotional, and social domains, through a variety of stimulus-rich movement experiences. Emphasis was placed on strengthening perception–action coupling by manipulating task, environmental, and individual constraints, a core strategy within the NLP framework. Sessions were designed to promote exploration, autonomous decision-making, and adaptive responses, rather than repetition of prescriptive motor patterns (full protocol available on Zenodo, https://doi.org/10.5281/zenodo.17312478).

Initial Phase (Activation, 10–15 min): Each session began with play-based motor exercises within open and variable environments, incorporating perceptual and spatiotemporal constraints. These tasks encouraged children to engage in self-regulation, motor adaptation, and exploratory movement. The goal was not to reproduce a single correct motor gesture, but to find individualised, functional solutions to motor challenges. Activities in this phase aimed to activate attention, perception, emotional engagement, and divergent thinking, while promoting global mobility.

Central Phase (Motor and Cognitive Engagement, 30 min): In this main phase, cognitive-motor exercises were introduced, characterised by a gradual progression of difficulty calibrated to the age group of the participants. These activities were designed to integrate two key objectives: Motor objective: to promote the acquisition and refinement of fundamental motor skills, with particular attention to coordinative and conditional components, as well as the development of gross motor abilities. Cognitive objective: to stimulate cognitive processes such as selective attention, inhibitory control, divided attention, visual discrimination, decision-making, self-organisation, and visual processing speed. From this perspective, the proposed games, often structured in cooperative or competitive formats between two or more teams, aimed to foster social and interactive dimensions while requiring children to continuously adapt their behaviour according to the task, role, and environmental conditions. This approach was intended to promote dynamic and context-based learning, consistent with the principles of NLP.

Final Phase (Relaxation and Verbalisation, ~5 min): The session concluded with a reflective phase aimed at promoting psychophysical recovery and emotional well-being. This stage provided a calm environment in which children could express their experiences through words, gestures, or silence. The emphasis here was on bodily metacognition, encouraging children to think about and reflect on their own motor actions. This aligns with a subject-centred pedagogical approach, helping to consolidate learning, enhance self-awareness, and facilitate the transition back to the classroom context.

### 2.3. Instruments

Knowledge and Informed Consent Questionnaire (PDPAR) [49]. The first instrument utilised was an introductory questionnaire, administered to parents following the provision of informed consent. Based on the Physical Activity Questionnaire for Children (PDPAR) framework, this tool was used to collect demographic and contextual information regarding the participants. The variables included age, gender, engagement in extracurricular sports activities, and the average number of hours per week spent in such activities.

Test of Gross Motor Development—3rd edition (TGMD-3). To assess children’s gross motor competence, the Italian version of the Test of Gross Motor Development, Third Edition (TGMD-3) was employed [50,51,52]. The assessments were conducted in school gyms during school hours, with physical education teachers present. Two trained physical education specialists simultaneously observed and scored each child’s performance in real time following the standard TGMD-3 protocol. To minimise potential bias, assessors were kept unaware of group allocation whenever feasible: evaluations were conducted outside the intervention context, and no information indicating whether a child belonged to the experimental or control group was provided. In situations where full blinding was not possible, due to the practical constraints of a school-based stud, assessments were carried out by examiners who were not involved in delivering the intervention, thereby reducing the risk of detection bias. Inter-rater reliability between the two observers was high, with an intraclass correlation coefficient (ICC[1,2]) = 0.92 (95% CI = 0.88–0.95) for the total TGMD-3 score, indicating excellent agreement.

This standardised instrument is designed for children aged 3 to 11 years and evaluates two core motor domains: locomotor skills, including running, galloping, jumping, hopping, horizontal jumping, and sliding; ball control skills, including two-handed strike, one-handed strike, dribbling, catching, kicking, overhead throw, and underhand throw. Each skill is assessed using a criterion-based checklist. Performance is scored with 1 point for each criterion met and 0 points for unmet criteria. Each child performed three trials: one practice trial followed by two formal trials. Only the scores from the two formal trials were recorded for evaluation. Performances were observed and assessed based on the qualitative performance criteria outlined for each TGMD-3 skill. The individual item score is derived from the sum of performance criteria across both formal trials. The scores from both subtests are combined to produce an overall Gross Motor Index (GMI). The administration of the TGMD-3 takes approximately 20 min per child [53]. 

WITTY SEM (Microgate, Bolzano). The Witty SEM system is a computerised platform designed to assess and enhance motor-cognitive skills such as attention, reaction time, agility, coordination, and visual processing speed. The Witty SEM tasks were computer-administered, with all performance indicators (reaction times and number of correct responses) recorded automatically by the system software. The examiner’s involvement was limited to starting each trial and ensuring that the child was correctly positioned. Because outcome scoring was fully automated and did not require subjective interpretation, assessor blinding was not necessary for these measures. This procedure substantially reduced the risk of detection bias for the cognitive outcomes.

In this study, Witty SEM was used to measure two key domains of executive function: divided attention and visual processing speed. The following tests were selected for this purpose: Cognitive Test 1—Divided Attention: This test evaluates the ability to simultaneously process and respond to multiple visual stimuli, requiring the distribution of attentional resources and cognitive flexibility. Level 1 presents two stimuli on LED modules placed in front of the participant. The child is required to detect and respond to specific target stimuli (e.g., coloured lights or symbols) by touching the corresponding module. This task assesses attentional allocation and parallel processing abilities. Level 2 increases complexity by introducing a greater variety of stimuli, shorter presentation times, and the presence of distractors, thereby challenging inhibitory control and cognitive flexibility. Outcome measures included reaction time and response accuracy, which serve as indicators of distributed attentional control. Cognitive Test 2—Hawk Eye: The Hawk Eye test is designed to assess visuoperceptual discrimination and decision-making speed in the presence of multiple competing stimuli. Level 1 presents a simple target stimulus among a limited number of distractors. The child must rapidly identify the target and respond appropriately. Level 2 introduces increased task difficulty by varying the number of distractors, stimulus presentation speed, and the required level of perceptual discrimination. This task evaluates abilities related to visual attention, stimulus discrimination, perceptual integration, and visuomotor coordination. As with the previous test, outcome measures included accuracy and average reaction time, both critical indicators of cognitive efficiency under time pressure [54].

All dependent variables were coded so that higher scores consistently represented better performance across measures (i.e., greater accuracy, faster responses, and higher Gross Motor Index [GMI] values).

Study registration and protocol. This study was conducted within a broader research programme on motor–cognitive development in primary school children. Owing to its quasi-experimental the study was not prospectively registered in a clinical trials registry. To ensure transparency and facilitate replication, the full study protocol—including lesson plans, fidelity procedures, and assessment methods—was made publicly available on Zenodo (https://doi.org/10.5281/zenodo.17312478).

### 2.4. Statistical Analysis

All statistical analyses were performed using IBM SPSS Statistics version 18.0 (SPSS Inc., Chicago, IL, USA). Baseline demographic and motor characteristics were collected for all participants, including age, sex, extracurricular physical activity (hours per week), and Gross Motor Index (GMI). All children attended the same school and, in line with the school curriculum, received exactly 2 h per week of structured physical education lessons. Baseline equivalence between groups was examined prior to testing intervention effects. Age was analysed using a two-way ANOVA with Group (control vs. experimental) and Sex (male vs. female) as fixed factors; this allowed the assessment of main effects and potential Group × Sex interactions. Sex distribution was compared between groups using a χ^2^ test. Group differences in extracurricular physical activity hours were evaluated using independent-samples t-tests and checks for Group × Sex interactions. No significant differences were observed for age, sex distribution, or extracurricular physical activity. These analyses confirmed that the experimental and control groups were comparable at baseline. Intervention effects were evaluated using two-way mixed-design ANCOVAs, with Group (experimental vs. control) as the between-subjects factor and Time (T1 vs. T2) as the within-subjects factor. Sex and Gross Motor Index (GMI) were included as covariates to improve the precision of the estimates. Although sex distribution did not differ between groups at baseline, sex is known to influence motor competence and motor–cognitive interaction in childhood [52,55], and adjusting for it reduces unexplained variance. GMI was included because baseline motor proficiency is theoretically and empirically linked to children’s performance in attention, visuomotor integration, and perceptual–motor tasks; controlling for GMI allows a clearer estimation of intervention-specific effects independent of initial motor ability.

Assumption checks were conducted prior to all analyses. Normality of residuals was examined using Shapiro–Wilk tests and Q–Q plots. Homogeneity of variances was evaluated through Levene’s test, and homogeneity of regression slopes was verified by testing the Group × Covariate interaction for each model. Linearity between covariates and outcomes was confirmed using scatterplots and partial regression plots. All assumptions were met. Minor deviations from normality in reaction-time variables were examined with bootstrapped ANCOVAs (1000 samples), which produced results equivalent to the original models, confirming the robustness of the findings. All tests were two-tailed with α = 0.05.

To quantify the magnitude of intervention effects, partial eta-squared (*η^2^ₚ*) was calculated for each group × time interaction. Cohen’s *d* values were computed from adjusted post-test means, using the pooled baseline standard deviation as the denominator. The formulas applied were:

$$\eta_{p}^{2}=\frac{F\times df_{1}}{F\times df_{1}+df_{2}},d=\frac{M_{adj,EG}-M_{adj,CG}}{SD_{pooled,baseline}}$$

Effect sizes followed Cohen’s interpretive thresholds: small (*d* ≈ 0.20; *η^2^ₚ* ≈ 0.01), medium (*d* ≈ 0.50; *η^2^ₚ* ≈ 0.06), and large (*d* ≥ 0.80; *η^2^ₚ* ≥ 0.14) [56]. For descriptive comparison with recent motor–cognitive intervention literature, classification thresholds proposed by Hopkins were also reported [57]. Because four cognitive outcomes were analysed (Divided Attention L1, Divided Attention L2, Hawk Eye L1, Hawk Eye L2), Type-I error inflation was addressed by applying the False Discovery Rate (FDR) correction using the Benjamini–Hochberg procedure [58]. Both unadjusted *p*-values (*p*) and FDR-adjusted *p*-values (pFDR) are reported, and all group × time interaction effects remained significant following correction. All statistical tests were two-tailed, and significance was set at *p* < 0.05.

Because all eight classes belonged to a single school, potential clustering effects were evaluated by estimating the intraclass correlation coefficients (ICCs) for each outcome. The estimated ICCs ranged from 0.02 to 0.04, indicating that less than 5% of the total variance was attributable to class-level nesting. Therefore, individual-level analyses were retained as the main analytical strategy. Robustness checks using cluster-robust standard errors confirmed the same pattern of results.

## 3. Results

All children in both the intervention and control groups attended at least 95% of the scheduled lessons. No adverse or unexpected events occurred during the 16-week intervention, and no participant withdrew due to injury, discomfort, or other safety concerns. The final sample consisted of 165 children (67 in the control group and 98 in the experimental group), with a mean age of 7.21 years (SD = 0.93). In the control group (CG; *n* = 67), 28 boys (42%) and 39 girls (58%) participated (mean age = 7.28 ± 1.02 years). In the experimental group (EG; *n* = 98), 44 boys (45%) and 54 girls (55%) took part (mean age = 7.31 ± 0.94 years).

Baseline characteristics are reported in Table 1. All children received 2 h per week of structured school-based physical education, ensuring consistent exposure across classes. No significant differences were found between groups in age (*p* = 0.842), sex distribution (*p* = 0.611), or extracurricular physical activity hours (*p* = 0.398). A two-way ANOVA confirmed that baseline age did not differ by Group (*p* > 0.80), by Sex (*p* > 0.50), nor through a Group × Sex interaction (*p* > 0.60). Similarly, extracurricular physical activity hours showed no significant main effects of Group or Sex and no interaction effects. These analyses confirm that the experimental and control groups were well balanced at baseline with respect to age, sex distribution, and physical activity behaviour.

The analysis presents several General Linear Model (GLM) models with within- and between-subjects factors. Sex and GMI were included as covariates in the ANCOVA models but did not exert significant effects on any of the outcomes (*p* = 0.911 and *p* = 0.969, respectively). The results therefore focus on the main effects of group, time, and their interaction. All *p*-values were corrected for multiple comparisons using the FDR procedure [58], and for each outcome both unadjusted *p*-values (*p*) and FDR-adjusted values (pFDR) are reported. All group × time interaction effects remained significant after correction. Table 2 presents descriptive statistics (means and standard deviations) for each test at pre-Test (T1) and post-Test (T2), along with effect sizes and ANCOVA results.

Across outcomes, both partial eta-squared (η^2^ₚ) and Cohen’s *d* values were computed to quantify the magnitude of intervention effects. The group × time interactions ranged from small-to-medium for the Divided Attention tasks (η^2^ₚ ≈ 0.04; *d* = 0.42–0.58) too large for the Hawk Eye tasks (η^2^ₚ = 0.12–0.15; *d* = 0.88–1.11), as showed in Table 3.

**Figure 2:**
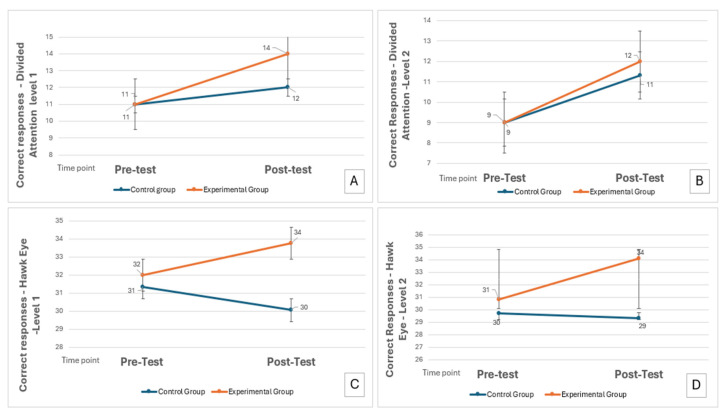
Correct responses WITTY SEM: (**A**) Correct responses—Test Divided Attention, Level 1; (**B**) Correct responses—Test Divided Attention, Level 2; (**C**) Correct responses—Test Hawk Eye Level 1; (**D**) Correct responses—Test Hawk Eye Level 2.

A statistically significant group × time interaction was found for Divided Attention Level 1, F (1161) = 6.219, *p* = 0.014 (pFDR = 0.014), with a medium effect size (d = 0.58). The experimental group (EG) improved from a mean score of 11.61 ± 1.7 at baseline to 13.20 ± 1.15 post-intervention. The control group (CG) improved from 11.64 ± 1.37 to 12.29 ± 0.75. These results indicate a greater benefit for the intervention group (see Figure 2A). Gender and GMI were not significant covariates (*p* ≥ 0.05).

Similarly, a significant interaction emerged for Divided Attention Level 2, F (1162) = 6.188, *p* = 0.014 (pFDR = 0.014), with a medium effect size (d = 0.42). The experimental group improved from 9.29 ± 2.24 at T1 to 11.99 ± 1.08 at T2, while the control group progressed from 9.32 ± 2.01 to 11.29 ± 0.95. This suggests greater attentional improvements in more complex conditions for the intervention group (see Figure 2B). No significant covariate effects were observed.

For the Hawk Eye Level 1 test, the experimental group showed an improvement from 32.45 ± 3.23 to 33.75 ± 2.03, while the control group declined from 31.35 ± 2.8 to 29.92 ± 1.90, resulting in a significant interaction: F (1162) = 21.432, *p* < 0.001 (pFDR = 0.002), with a large effect size (d = 0.88). These results highlight the effectiveness of the intervention in enhancing visuoperceptual discrimination and decision-making speed (see Figure 2C).

A similar pattern was observed for Hawk Eye Level 2, where the experimental group improved significantly from 30.85 ± 3.02 to 33.80 ± 1.6, while the control group showed no improvement (29.77 ± 2.98 at T1 to 29.37 ± 2.42 at T2). The ANCOVA revealed a strong interaction effect, F (1162) = 28.554, *p* < 0.001 (pFDR = 0.002), with a very large effect size (d= 1.11) again favouring the intervention (see Figure 2D).

For all Witty SEM and TGMD-3 measures, higher scores indicate better performance, including improved attention, faster visual processing, and greater gross motor competence. Table 4 provides a structured overview of the pre–post changes in attentional and visual-processing outcomes for both the control and experimental groups. By presenting pre-test scores, post-test scores, and the corresponding mean change (Δ), the table enables a clear comparison of performance trajectories across groups and task types.

## 4. Discussion

The primary finding of this study is that the NLP-based intervention produced significantly greater improvements in cognitive functions, specifically attentional performance and processing speed, among children in the experimental group compared with those in the control group. Intervention fidelity was excellent, with 100% of planned sessions delivered and participant attendance exceeding 95%, indicating strong adherence and supporting the internal validity of the study. No adverse events were reported, confirming the safety and feasibility of implementing nonlinear pedagogical motor programmes within primary school settings. The results demonstrated positive effects of the intervention across multiple dimensions of executive functioning. Improvements were observed in divided attention, visuoperceptual discrimination, and decision-making under pressure, as measured by both levels of the Divided Attention (DA) and Hawk Eye (HE) tests. Performance enhancements were marked in the experimental group (EG), manifesting consistently across tasks of varying difficulty levels. A key methodological strength of the study lies in the baseline equivalence between groups. No significant differences were found between the EG and CG with regard to sex or initial GMI. This homogeneity enhances the internal validity of the results and supports a causal interpretation of the intervention’s effects. In the Divided Attention test, both levels revealed significantly greater improvements in the EG compared to the CG. While both groups showed some progress, the magnitude of improvement was notably higher in the experimental group, indicating that the intervention specifically enhanced children’s ability to selectively attend to and manage multiple visual stimuli, an essential executive function in cognitively demanding tasks. The Hawk Eye test, which requires high-level perceptual integration, visuo-motor coordination, and rapid decision-making, further highlighted the benefits of the intervention. The experimental group showed marked gains, particularly at the second level of difficulty, where they improved by approximately three points on average, while the control group showed a slight decline. These findings suggest that the intervention not only supported basic attentional processes but also contributed to higher-order cognitive flexibility and adaptive functioning under increased task complexity. Taken together, these results support the hypothesis that play-based physical education activities, when designed according to NLP and ecological-dynamic principles, can meaningfully promote the development of executive functions in school-age children. Significantly, the improvements observed at more complex test levels suggest the possibility of a transfer effect, that is, the generalisation of cognitive benefits to novel or more demanding contexts. This aligns with recent research suggesting that movement-based interventions embedded with cognitive challenges can accelerate the development of core cognitive skills [59,60,61,62,63].

A major and original outcome of the present study is its demonstration that NLP-informed physical education sessions, delivered over a 16-week period, produced measurable cognitive benefits above and beyond the standard curriculum. While prior research has indicated that physically active learning may improve academic performance, classroom behaviour, and general engagement, previous meta-analyses have found mixed or inconclusive evidence regarding the direct impact of such interventions on cognitive function [64,65,66,67]. In contrast, this study contributes novel empirical evidence that physical activity, when implemented through a structured, cognitively enriched, and learner-centred pedagogy, can lead to improvements in specific cognitive domains such as attention, executive control, working memory, and visual processing. These enhanced cognitive abilities may represent a mechanistic pathway through which physically active learning contributes to academic achievement and educational outcomes. Previous research has indicated that participation in physical education (PE) interventions may lead to a more efficient use of neural resources associated with executive functions, as evidenced by increased neural activity in brain regions involved in attention and working memory [68,69]. When physical activity is coupled with cognitive demands—such as through problem-solving tasks, rule variations, and decision-making scenarios embedded within movement contexts—children’s executive functions are more actively engaged. These activities are believed to support information processing, working memory, and memory consolidation [60,70,71], providing a neurocognitive basis for the improvements observed in this study.

The distinctive contribution of this study resides in its explicit examination of cognitive outcomes emerging from a nonlinear, exploration-driven educational approach. The findings emphasise that intentional pedagogical design plays a critical role in promoting holistic child development, not only enhancing motor competence but also shaping attention, inhibition, and flexible thinking. Consistent with the NLP framework [24], learning is understood as an emergent property of the continuous interaction between the learner and their environment [38,41]. Environments rich in perceptual stimuli, variability, and uncertainty foster adaptive behaviours and demand real-time cognitive engagement [28,35,44]. Such contexts require children to continuously adjust their responses, thereby activating inhibitory control, cognitive flexibility, and working memory, core components of executive functioning [31,72]. The absence of rigid motor patterns and the inclusion of multi-solution tasks promote not only physical autonomy but also enhance sustained and selective attention by increasing intrinsic motivation and personal engagement.

From an educational perspective, these findings support the importance of designing motor learning experiences that are exploratory rather than prescriptive, with built-in elements of novelty, challenge, and self-directed decision-making. Such an approach aligns with the principles of nonlinear pedagogy and has the potential to improve both motor competence and cognitive outcomes in school-aged children [23,73,74,75]. The implications for curriculum design are significant. Incorporating intentional and variable motor activities into physical education, as outlined in the Italian National Curriculum Guidelines, may serve as an effective strategy for enhancing children’s executive functions, particularly attention, processing speed, and adaptive learning skills. These cognitive gains may, in turn, support improved academic performance, self-regulation, and learning motivation, critical contributors to overall educational success.

Despite the promising findings, this study has several limitations. First, although the sample size was adequate for detecting medium intervention effects, it limits the generalisability of the results to broader populations and other age groups. Second, although the intervention lasted 16 weeks, it is possible that a longer duration may have produced more sustained or amplified cognitive and motor benefits. The two groups differed in size (98 vs. 67 participants); however, this imbalance reflected natural class enrolment patterns rather than differential attrition. The ANCOVA models used are robust to moderate group-size differences when homogeneity of variance is satisfied, an assumption confirmed in our analyses, and bootstrapped models produced consistent results, indicating that unequal sample sizes did not bias the findings.

The study was also limited in its ability to control contextual and environmental factors, such as classroom dynamics, extracurricular activities, or socioeconomic background, which may independently influence developmental outcomes. Socioeconomic status (SES) indicators could not be collected due to national school privacy regulations, preventing the examination of SES as a potential moderator of intervention effects.

Future research would benefit from the inclusion of SES measures, larger and more demographically diverse samples, and longitudinal designs capable of assessing the durability of cognitive and motor improvements. Further studies should also incorporate more fine-grained assessments of executive functions (e.g., inhibition, shifting, updating) and qualitative methods (e.g., classroom observations, teacher or parent feedback) to deepen understanding of implementation fidelity and children’s engagement. Such work would help consolidate and extend the growing evidence supporting nonlinear, cognitively enriched motor programs in primary education.

## 5. Conclusions

The primary aim of this study was to evaluate the effectiveness of a school-based educational intervention designed to promote the development of higher-order cognitive skills in primary school children. The intervention incorporated core principles of NLP into physical education, with the objective of stimulating cognitive abilities and attentional processes through enriched motor activities. The findings suggest that cognitive development can be significantly enhanced through physical activity, provided that the activities are purposefully structured to include variability, cognitive challenge, and learner autonomy. Compared to traditional PE lessons, the NLP-based approach demonstrated superior outcomes in attention, processing speed, and decision-making under pressure. The encouraging outcomes of this study call for further research involving larger cohorts and longer intervention durations. They also lend support to the recommendation that schools implement at least two hours of physical education weekly, embedded within dynamic, varied, and cognitively stimulating learning contexts. Such an approach not only supports motor competence but also facilitates the development of executive functioning, thereby contributing to the holistic development of children during a critical period of growth. Ultimately, physical education that incorporates continuous cognitive stimulation appears to provide synergistic benefits for both motor and cognitive development, reinforcing the importance of integrating movement and thinking within early educational practice.

## Figures and Tables

**Table 1:** Baseline characteristics.

Variable	Control Group (CG)	Experimental Group (EG)	*p*-Value
*n*	67	98	—
Age, years (M ± SD)	7.28 ± 1.02	7.31 ± 0.94	0.842
Sex, n (%)			0.611
Male	28 (42%)	44 (45%)	
Female	39 (58%)	54 (55%)	
Extracurricular PA hours/week (M ± SD)	2.71 ± 1.52	2.96 ± 2.18	0.398
School-based PE hours/week	2.0 for all children	2.0 for all children	—

**Table 2:** ANCOVA Results by Group and Timepoint.

Test	Group Type	Pre-Test		Post-Test		Main Effect Group	
		Mean	SD	Mean	SD	Effect Size (Cohen’s d)	Analysis of Covariance
**Divided Attention** **Level 1**	Control Group	11.6	1.3	12.2	0.75	0.58	F (1161) = 6219, *p* = 0.014 (pFDR = 0.014)
	Experimental Group	11.6	1.7	13.1	1.15		
**Diveded Attention** **Level 2**	Control Group	9.3	2.01	11.2	0.95	0.42	F (1162) = 6188, *p* = 0.014 (pFDR = 0.014)
	Experimental Group	9.2	2.2	11.9	1.08		
**Hawk Eye** **Level 1**	Control Group	31.3	2.8	29.9	1.9	0.88	F (1162) = 21,432, *p* < 0.001 (pFDR = 0.002)
	Experimental Group	32.4	3.2	33.7	2.03		
**Hawk Eye** **Level 2**	Control Group	29.7	2.9	29.3	2.4	1.11	F (1162) = 28,554, *p* < 0.001 (pFDR = 0.002)
	Experimental Group	30.8	3.02	33.7	1.6		

**Table 3:** Adjusted post-test means, effect sizes, and ANCOVA results for cognitive outcomes.

Test	F (1, df_2_)	*p*	η^2^ₚ	d	Interpretation
**Divided Attention L1**	6.219 (1, 161)	0.014	0.04	0.58	Medium
**Divided Attention L2**	6.188 (1, 162)	0.014	0.04	0.42	Small–Medium
**Hawk Eye L1**	21.432 (1, 162)	<0.001	0.12	0.88	Large
**Hawk Eye L2**	28.554 (1, 162)	<0.001	0.15	1.11	Large–Very Large

**Table 4:** Change scores (Δ = Post-test − Pre-test) for all outcome measures.

Outcome	Group	Pre-Test M (SD)	Post-Test M (SD)	Δ Mean (SD)	Interpretation
**Divided Attention Level 1**	Control	11.6 (1.3)	12.2 (0.75)	+0.6 (1.1)	Small improvement
	Experimental	11.6 (1.7)	13.1 (1.15)	+1.5 (1.3)	Moderate improvement
**Divided Attention Level 2**	Control	9.3 (2.01)	11.2 (0.95)	+1.9 (1.1)	Moderate improvement
	Experimental	9.2 (2.2)	11.9 (1.08)	+2.7 (1.4)	Greater improvement
**Hawk Eye Level 1**	Control	31.3 (2.8)	29.9 (1.9)	−1.4 (1.2)	Decline
	Experimental	32.4 (3.2)	33.7 (2.03)	+1.3 (1.5)	Improvement
**Hawk Eye Level 2**	Control	29.7 (2.9)	29.3 (2.4)	−0.4 (1.0)	No change
	Experimental	30.8 (3.02)	33.7 (1.6)	+2.9 (1.5)	Strong improvement

## Data Availability

The original data presented in the study are openly available on Zenodo at: https://doi.org/10.5281/zenodo.17312478.

## Abbreviations

The following abbreviations are used in this manuscript:

NLP	NonLinear Pedagogy
PE	Physical Education
EG	Experimental Group
CG	Control Group
GMI	Gross Motor Index
DA	Divided Attention
HE	Hawk Eye
